# Vibrational Mode-Specific Dynamics of the OH + C_2_H_6_ Reaction

**DOI:** 10.1021/acs.jpca.3c04328

**Published:** 2023-08-24

**Authors:** Balázs Gruber, Viktor Tajti, Gábor Czakó

**Affiliations:** MTA-SZTE Lendület Computational Reaction Dynamics Research Group, Interdisciplinary Excellence Centre and Department of Physical Chemistry and Materials Science, Institute of Chemistry, University of Szeged, Rerrich Béla tér 1, Szeged H-6720, Hungary

## Abstract

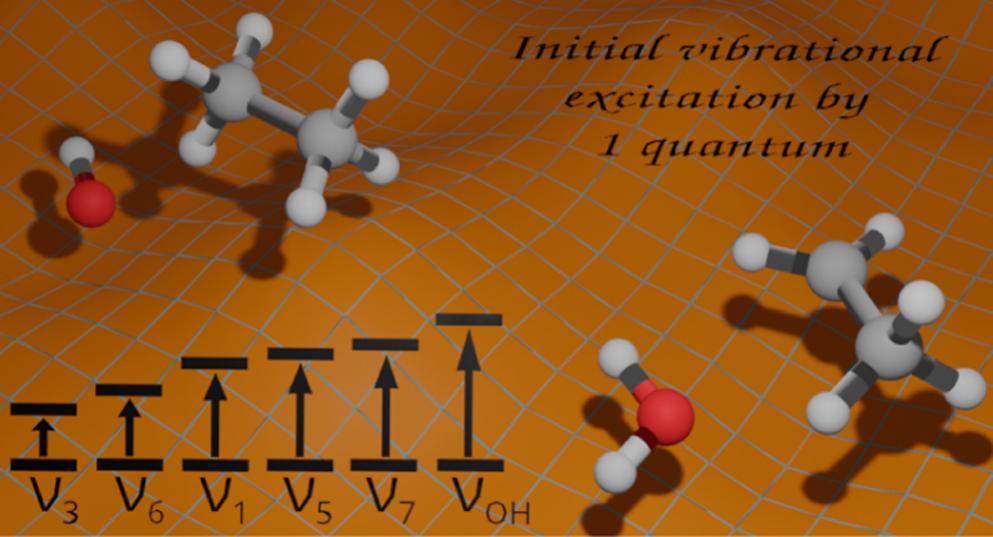

We investigate the effects of the initial vibrational excitations on the dynamics of the OH + C_2_H_6_ → H_2_O + C_2_H_5_ reaction using the quasi-classical trajectory method and a full-dimensional analytical ab initio potential energy surface. Excitation of the initial CH, CC, and OH stretching modes enhances, slightly inhibits, and does not affect the reactivity, respectively. Translational energy activates the early-barrier title reaction more efficiently than OH and CC stretching excitations, in accord with the Polanyi rules whereas CH stretching modes have similar or higher efficacy than translation, showing that these rules are not always valid in polyatomic processes. Scattering angle, initial attack angle, and product translational energy distributions show the dominance of direct stripping with increasing collision energy, side-on OH and isotropic C_2_H_6_ attack preferences, and substantial reactant–product translational energy transfer without any significant mode specificity. The reactant vibrational excitation energy of OH and C_2_H_6_ flows into the H_2_O and C_2_H_5_ product vibrations, respectively, whereas product rotations are not affected. The computed mode-specific H_2_O vibrational distributions show that initial OH excitation appears in the asymmetric stretching vibration of the H_2_O product and allow comparison with experiments.

## 1 Introduction

Understanding and controlling energy transfer in polyatomic reactive chemical systems are important goals of modern reaction dynamics studies. The energy efficiency of a chemical reaction depends on the form of energy invested in the reactants. In the case of a bimolecular reaction, one may add translational energy or excite the internal rotational–vibrational degrees of freedom of the colliding molecules. The Polanyi rules^1^ predict that collision energy plays a more important role in activating the early-barrier (reactant-like transition state) reactions, whereas vibrational excitations promote the late-barrier reactions more efficiently. These simple rules can be straightforwardly applied for reactions of atoms with diatomic molecules as Polanyi did;^1^ however, the picture becomes more complicated in the case of polyatomic reactants, where multiple vibrational modes exist.^2–4^ In the latter case, vibrational mode-specific effects can be observed; some modes may enhance reactivity, others inhibit the reaction or behave as spectators. Such mode-specific effects were first found for the H + H_2_O/HOD reactions^5–8^ and then other atom + water and methane reactions became the benchmark systems to study mode-specific polyatomic reactivity.^2–4,9–16^ In 2013, a quantification of the Polanyi rules with a sudden vector projection model was proposed by Jiang and Guo,^17^ which method was successfully applied to many of the above reactions.^18^ Recently, the attention has also turned toward even more complicated 7- and 9-atomic systems, such as atom + ethane^19–21^ and methanol^22,23^ as well as hydrogen-halide + ethyl reactions.^24,25^

In the present study, we continue the mode-specific investigations with the 10-atomic OH + C_2_H_6_ → H_2_O + C_2_H_5_ reaction. Early experimental and theoretical work mostly focused on the kinetics of the title reaction.^26–28^ Nevertheless, mode-specific vibrational distributions for the H_2_O product were also probed experimentally by Butkovskaya and Setser in 2003.^29^ The theoretical investigations on the OH + C_2_H_6_ reaction were restricted to the use of transition-state theory until 2020,^27,28^ when Rangel et al.^30^ reported a full-dimensional valence bond-molecular mechanics potential energy surface (PES) allowing kinetics and dynamics simulations for the H-abstraction process. In the same year, we also started to investigate the title reaction using high-level ab initio methods such as CCSD(T)-F12b, CCSDT(Q), and MRCI showing that, besides the H_2_O + C_2_H_5_ channel, hydrogen- and methyl-substitution can also occur at higher collision energies resulting in the H + C_2_H_5_OH and CH_3_ + CH_3_OH products, respectively.^31^ Based on the benchmark ab initio characterization of the stationary points of the multichannel OH + C_2_H_6_ reaction,^31^ in 2022 we developed a full-dimensional coupled-cluster-quality analytical PES, which allowed efficient dynamics investigations using the quasi-classical trajectory (QCT) method.^32^ The dynamics simulations for the ground-state OH(ν = 0) + C_2_H_6_(ν = 0) reaction showed that the reactivity of the hydrogen- and methyl-substitution channels is negligible besides hydrogen abstraction in the collision energy range of 10–50 kcal/mol. Following our previous work,^32^ here we perform mode-specific dynamics simulations for the OH + C_2_H_6_ → H_2_O + C_2_H_5_ reaction utilizing our analytical ab initio PES and the QCT method. The dynamics results obtained for different excited initial vibrational states at various collision energies provide new insights into the validity of the Polanyi rules for a reactive system with 24 vibrational degrees of freedom. Furthermore, we perform mode-specific vibrational analysis for the H_2_O product, allowing direct comparison with the experiments of Butkovskaya and Setser^29^ and following the state-to-state energy transfer in the title reaction. In Section 2, we describe the details of the dynamics simulations and product analysis, the results are given and discussed in Section 3, and the paper ends with a summary and conclusions in Section 4.

## 2 Computational Details

Quasi-classical simulations are performed for the OH + C_2_H_6_ reaction on a full-dimensional PES^32^ developed automatically using the Robosurfer program package.^33^ We are interested in examining the impact of exciting five vibrational modes of the ethane molecule and one vibrational mode of the hydroxyl radical with one quantum at the beginning of the QCT simulations and comparing these results with our previous work,^32^ where ground-state simulations were carried out.

The trajectories are computed at collision energies of 10, 20, 30, 40, and 50 kcal/mol. 85 000 simulations are run at the excitations of every single normal mode by performing 1000 trajectories at each variation of collision energy and impact parameter (the perpendicular distance of the velocity vectors of the reactants). All in all, 1000(trajectories) × 17(impact parameters) × 5(collision energies) × 6(normal modes) = 510 000 QCT simulations are studied in our present research. The spatial orientations of the reactants are randomly chosen and the initial distance of the center of masses of the OH and the C_2_H_6_ molecules is , where *x* = 18.90 bohr (10 Å) and the *b* impact parameter is modified between 0 and 8 bohr with 0.5 bohr steps. The trajectories are propagated until the largest atom–atom distance is greater than the greatest initial one by 1 bohr with a 0.0726 fs time step, which corresponds to 3 atomic units. The initial vibrational states of the OH and C_2_H_6_ molecules are prepared by standard normal-mode sampling.^34^

Integral cross sections (ICSs) (σ) are determined by using a *b*-weighted numerical integration of the opacity function [*P*(*b*)]
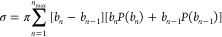
1where *n*_max_ represents the number of *b* intervals covering the range of [0, *b*_max_]. *b*_max_ is considered as the maximum impact parameter, where the reaction probability becomes zero. In this work, *b*_*n*_ = 0.5*n* bohr, where *n* = 0, 1, ..., *n*_max_. In order to check the statistical accuracy of the cross sections, we analyzed 500 and 1000 trajectories at each *b*, which resulted in the same σ values with only 1.5% average deviation. We introduce two different zero-point-energy (ZPE) constraints with regard to the products: soft and hard. Within the soft case, those trajectories are taken into account where the sum of the classical vibrational energies of the products is greater than the sum of their harmonic ZPEs. In the hard case, this restriction is considered separately for each product. The scattering angle distributions are calculated by binning the cosine of the angle (θ) of the relative velocity vectors of the center of masses of the reactants and products into ten equidistant bins between −1 and 1, where cos(θ) = −1 corresponds to backward scattering and cos(θ) = 1 means forward scattering. Additionally, the initial attack angle distributions are determined for the reactants by binning the cosine of the angle (α) of the velocity vector of the center of mass of one of the reactants and an interatomic vector, which is chosen as the O–H bond for the hydroxyl radical and the C–C bond for the ethane molecule. Ten equidistant bins are also used in this case from −1 to 1. When cos(α) = −1, the OH radical goes toward the ethane molecule with its O-side. In the case of cos(α) = 1, the OH radical approaches the ethane molecule with a H atom. Rotational quantum numbers (*J*) of the product molecules are calculated by rounding the lengths of classical rotational angular momentum vectors (in atomic units) to the nearest integer values.

Mode-specific vibrational distributions for the H_2_O product are computed based on the procedure detailed in ref (35). In the first step, we determine the harmonic vibrational frequencies (ω_*k*_, *k* = 1, 2, and 3) and normal-mode eigenvectors for the water molecule optimized on the present potential energy surface utilizing normal-mode analysis. Then, we remove the angular momentum by modifying the velocities. After that, we determine the best overlap between the optimized structure and the actual geometry in the case of every reactive trajectory using an Eckart transformation. In the next step, the normal coordinates (*Q*_*k*_) and momenta (*P*_*k*_) are obtained from the mass-scaled Cartesian displacement coordinates and velocities with the help of the transformation matrix determined in the first step. Finally, the mode-specific harmonic vibrational energies (*E*_*k*_) and the integer vibrational quantum numbers are calculated as
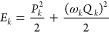
2
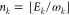
3where *n*_*k*_ is obtained by rounding down to the closest integer number. After that, we determined the vibrational distributions using the histogram binning technique. The following equation gives the probability of a unique vibrational state, **n** = (*n*_1_, *n*_2_, *n*_3_), at a specific impact parameter (*b*)
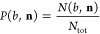
4where *N*(*b*, **n**) is the number of H_2_O molecules at state **n** and *N*_tot_ is the total number of trajectories at a given *b*.

## 3 Results and Discussion

**Figure 1:**
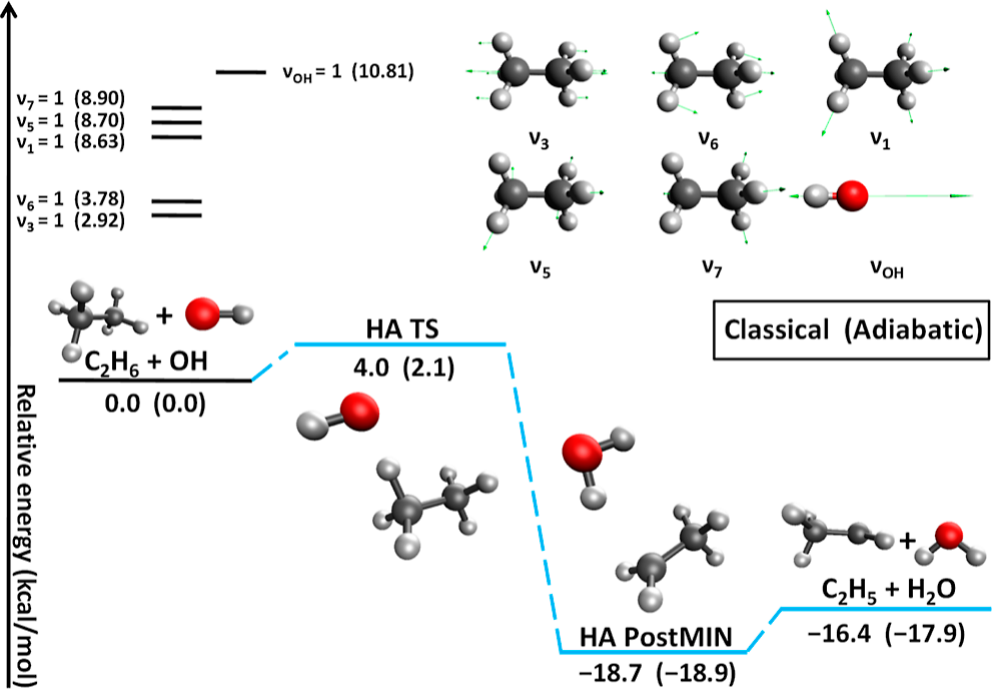
Normal-mode vibrations of C_2_H_6_ and OH investigated in the present study: ν_3_ = C–C stretching, ν_6_ = CH_3_ deformation, ν_1_ = symmetric CH stretching, ν_5_ = asymmetric CH stretching, ν_7_ = degenerate CH stretching, and ν_OH_ = OH stretching, where ν_*x*_ [*x* = 1, 3, 5, 6, 7] refer to the standard Mulliken notations as well as the schematic potential energy surface of the OH + C_2_H_6_ → H_2_O + C_2_H_5_ reaction showing the classical (adiabatic) relative energies of the stationary points calculated on the analytical PES.^32^

We examine the H-abstraction pathway of the OH + C_2_H_6_ reaction with the help of an analytical PES by exciting five normal modes of the ethane and one normal mode of the OH molecule as can be seen in Figure 1 including the schematic potential energy surface of the investigated reaction. To proceed with this reaction, it is necessary to invest a small amount of energy (4.0 kcal/mol classically and 2.1 kcal/mol adiabatically) to reach the transition state (HA TS) of the H-abstraction reaction, where the OH radical approaches the ethane molecule with an approximately 90° HOH angle. The process continues with a relatively deep (−18.7 kcal/mol classical and −18.9 kcal/mol adiabatic) postreaction minimum (HA PostMIN), where the OH radical rips off a H from the ethane molecule resulting in a structure, where the H atom of the OH radical is inverted closer to the C–C bond of the ethane molecule. In the final step, the H_2_O and C_2_H_5_ products are formed with an energy level (−16.4 kcal/mol classically and −17.9 kcal/mol adiabatically) below the reactants; thus, the reaction is exothermic.

The hydrogen- and methyl-substitution channels proceed over high classical (adiabatic) barriers of 43.0(41.4) and 37.7(36.0) kcal/mol (see ref (32) for the schematic PES), respectively, and their cross sections, in the case of the ground-state reaction, are only 0.030 and 0.024 bohr^2^, in order, at our highest collision energy (*E*_coll_) of 50 kcal/mol. Initial CH-, and somewhat surprisingly, CC stretching excitations increase the reactivity of the hydrogen-substitution channel by a factor of 2 and CC stretching excitation enhances the methyl substitution by a factor of 3. However, even in the case of initial vibrational excitations, the cross sections of these high-barrier channels are still low and the enhancement factors have significant statistical uncertainties; thus, in the present work we focus on the H-abstraction channel, which has 3 orders of magnitude higher reactivity.

**Figure 2:**
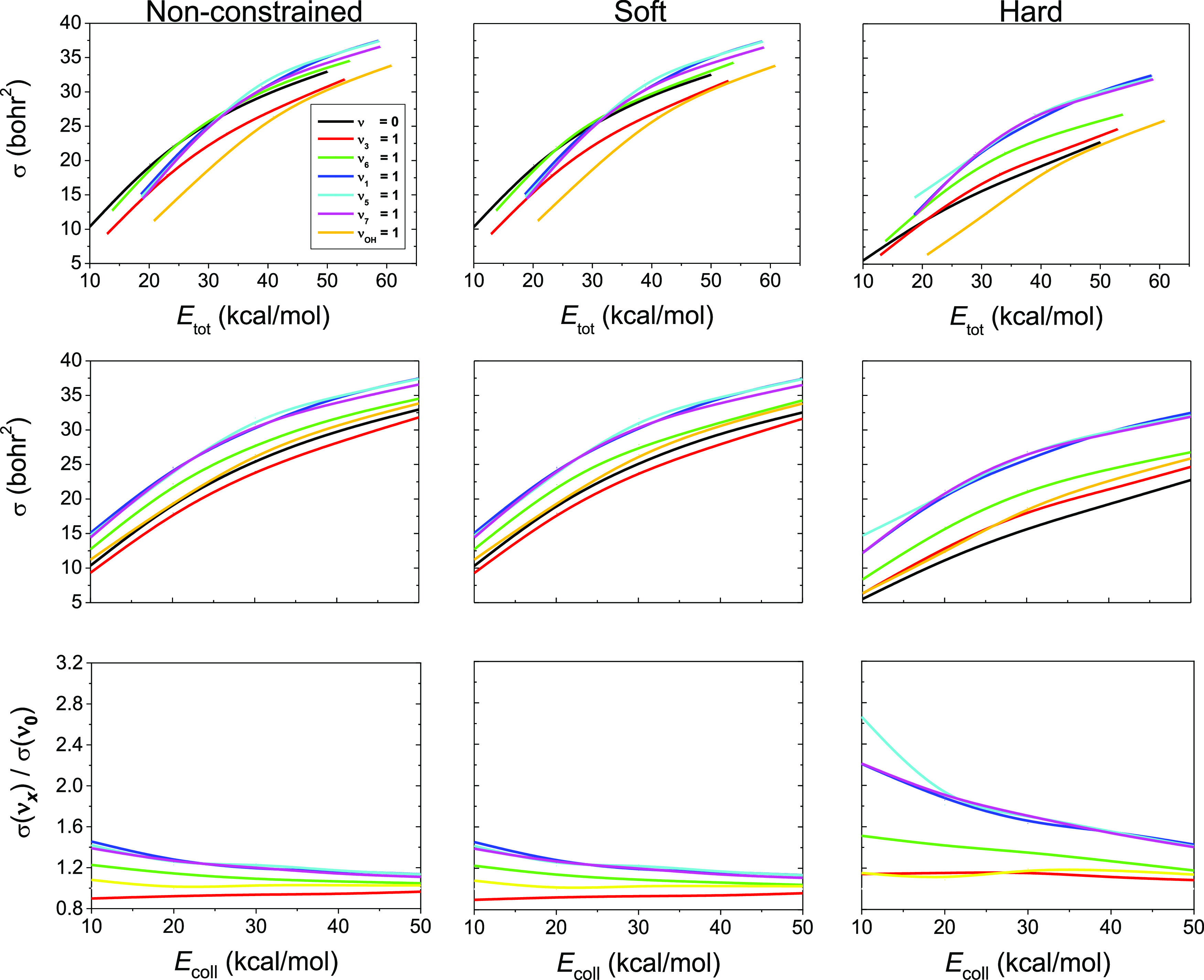
ICSs of the OH + C_2_H_6_ → H_2_O + C_2_H_5_ reaction as a function of total energy (collision energy + vibrational excition energy, upper panels) and collision energy (middle panels) as well as the ratios of ICSs of the OH(ν_OH_ = 0) + C_2_H_6_(ν_*x*_ = 1) and OH(ν_OH_ = 1) + C_2_H_6_(ν_*x*_ = 0), *x* = 3, 6, 1, 5, 7, reactions with respect to the OH(ν_OH_ = 0) + C_2_H_6_(ν = 0) unexcited reaction as a function of collision energy (lower panels) determined without as well as with soft and hard ZPE constraints.

Figure 2 shows the ICSs of the H-abstraction channel as a function of total (initial translational + vibrational) and collision energy as well as the ratios of the ICSs of the excited and unexcited initial states as a function of collision energy without and with soft and hard constraints. The reactivity increases in every case of normal-mode excitations with increasing total and collision energies. Observing the total energy dependence of the ICSs, in two cases of normal-mode excitations (ν_3_ and ν_OH_) we clearly get smaller reactivity compared to the unexcited reaction without or with soft constraint. With hard constraint, this phenomenon only refers to the case when the OH stretching is excited. The fact that OH stretching excitation does not significantly affect the reactivity is expected because the OH vibration is likely to be a spectator mode in this reaction. Similarly, there is one normal-mode excitation (ν_3_) in the case of examining the *E*_coll_ dependence of the ICSs where the reactivity does not exceed the unexcited one without as well as with soft constraint. Using a hard ZPE constraint, the reactivity is always larger in the case of every normal-mode excitation than the ground-state. The soft ZPE constraint has almost no effect on the reactivities, whereas in the case of the hard constraint, a significant decrease in the ICSs is noticeable. The most substantial enhancing effects are found for the CH stretching modes (ν_1_, ν_5_, and ν_7_) as expected because a CH bond breaks in the H-abstraction process. The total energy dependence of the reactivity for the different initial vibrational states allows checking the validity of the Polanyi rules^1^ in the title reaction. As Figure 1 shows, the HA TS is reactant-like, suggesting that translational energy is more efficient than vibrational excitation for enhancing the reactivity. This means that at a given total energy, the ground-state reaction, where *E*_coll_ is the largest, should have the highest reactivity. This clearly holds for ν_3_ = 1 (except hard constraint) and ν_OH_ = 1, but the other excited reactions show comparable or even higher (especially with hard constraint) reactivity with or than the ground-state reaction, contradicting the Polanyi rules. Seeing this unexpected finding, it is important to note that we did not find similar violation of the Polanyi rules for the similarly early-barrier F + C_2_H_6_ reaction.^21^ However, we also note that violation of these rules was reported for several other early-barrier systems such as F + H_2_O, HCl + OH, and OH + H_2_S.^36–38^

**Figure 3:**
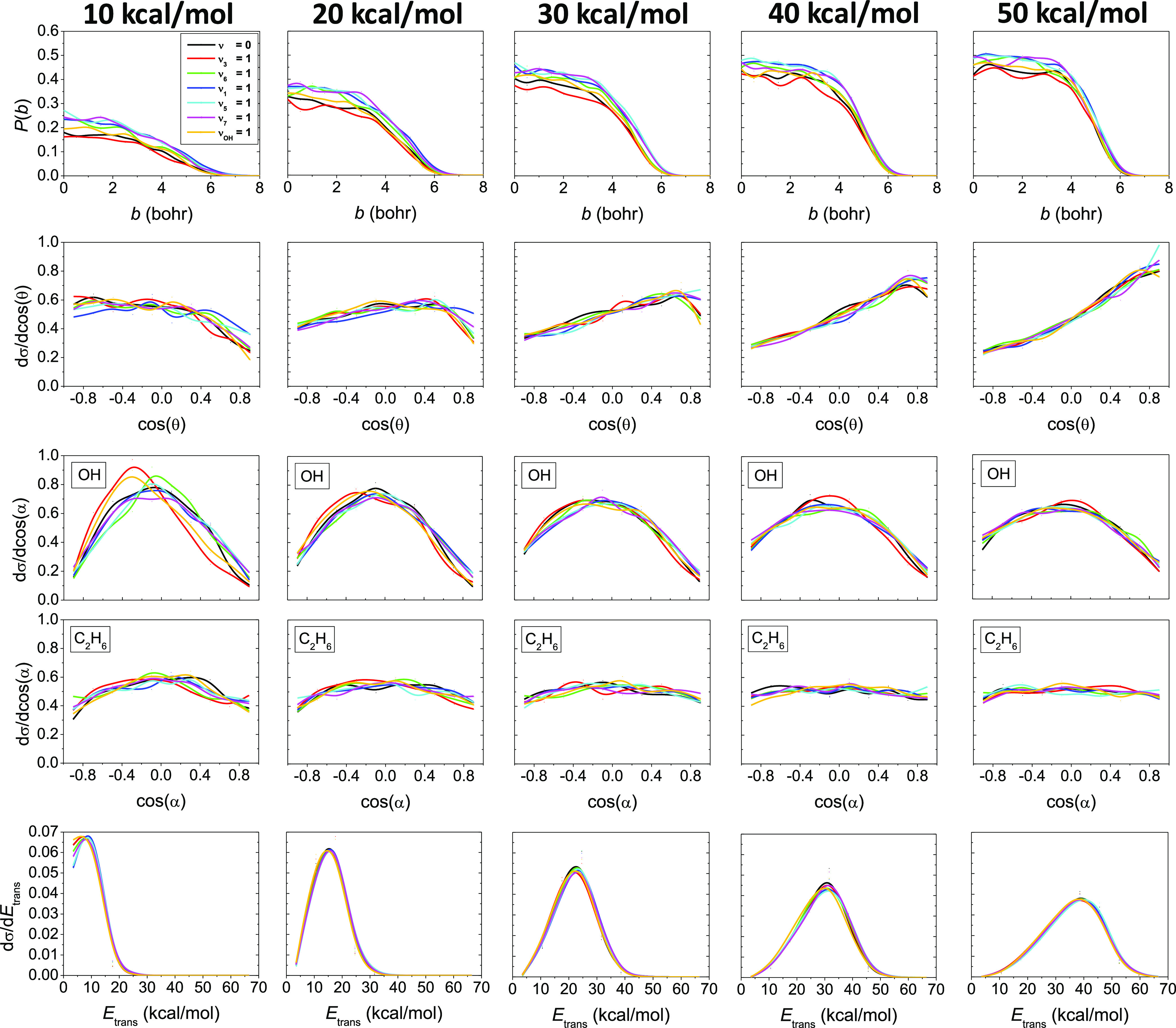
Visualized from up to down: reaction probabilities as a function of impact parameter, normalized scattering angle distributions, normalized initial attack angle distributions for the reactant OH and C_2_H_6_ molecules, and normalized product relative translational energy distributions for the OH(ν_OH_ = 0) + C_2_H_6_(ν_*x*_ = 0, 1), *x* = 3, 6, 1, 5, 7, and OH(ν_OH_ = 1) + C_2_H_6_(ν = 0) reactions at different collision energies.

The reaction probabilities as a function of impact parameter, the normalized product scattering angle distributions, the normalized initial attack angle distributions for the reactant OH and C_2_H_6_, and the product relative translational energy distributions are shown in Figure 3 at different collision energies. Opacity functions show that the maximum impact parameter is near 6.5 bohr without any *E*_coll_ or initial-state dependence. The probability of the reaction where the C–C stretching (ν_3_) of ethane molecule has 1 quantum vibrational excitation is always below the unexcited reaction, in accord with the ICSs. At *b* = 0, the reaction probability values get larger and larger with increasing *E*_coll_ until *E*_coll_ = 30 kcal/mol. After that, we cannot experience any further significant increase in the reaction probability, in accord with the shallower slope of the excitation functions. Excitation of the CH stretching modes (ν_1_, ν_5_, and ν_7_) clearly increases the reaction probabilities, again in accordance with the ICSs. As we have recently discussed in our previous work,^32^ the scattering angle distributions are connected to the shape of these opacity functions. At the lowest collision energy, the probabilities show a monotonic decrease with increasing impact parameter until approximately *b* = 4 and then the curves rapidly drop off. As the collision energy increases, the larger *b* values become preferred. Thus, the rebound mechanism, which favors smaller *b* values, dominates at lower collision energies. As the collision energy increases, the direct stripping mechanism, which prefers larger *b* values, become more and more pronounced. In accord with the shape of the opacity functions, backward scattering dominates at low collision energies, mostly at *E*_coll_ = 10 kcal/mol as a sign of the direct rebound mechanism, and as we go to higher collision energies, the forward scattering becomes more and more typical as a sign of direct stripping. Moreover, little isotropic scattering angle distributions are shown, mostly at 10 and 20 kcal/mol collision energies, which indicates an indirect character for the investigated reaction, promoting complex formation via the process. Significant mode-specific feature cannot be discovered in the case of the scattering angle distributions. Initial attack angle distributions show that the OH radical slightly prefers to attack with its O-side over H-side because an O–H bond is formed through the H-abstraction reaction. However, the typical way of attacking of the OH radical is the side-on attack. This kind of preference can be explained with the structure of the HA TS, where the H–O–H angle is almost 90°. The initial attack angle distributions of the OH radical show a small mode-specific character in the case of the initial vibrational excitation of the OH radical and the ν_3_ mode of ethane at 10 kcal/mol collision energy. However, as the collision energy increases, the mode-specific feature disappears from the distributions and they become more and more isotropic. The attack angle distributions of the C_2_H_6_ molecule show a slight preference for side-on attack at 10 and 20 kcal/mol collision energies, but as the collision energy increases the distributions become basically isotropic, suggesting that the C_2_H_6_ molecule behaves as a spherical object, like CH_4_. Mode-specific attitude does not appear in the C_2_H_6_ initial attack angle distributions. Looking at the relative translational energy distributions of the H_2_O and C_2_H_5_ products of the OH + C_2_H_6_ reaction, it is observable that the distributions become more and more broader and substantially shift toward the higher translational energies with increasing collision energy, which suggest efficient translational energy transfer between the reactants and products. Furthermore, one can notice that as the collision energy increases the peaks of the distributions go toward the highest available translational energies, which indicates the more direct behavior of the reaction as the collision energy increases, which is in good agreement with the scattering angle distributions. The relative translational energy distributions of the products do not show any noticeable vibrational mode-specific feature, indicating that the initial vibrational excitation energy mainly transfers into the internal degrees of freedom of the products.

**Figure 4:**
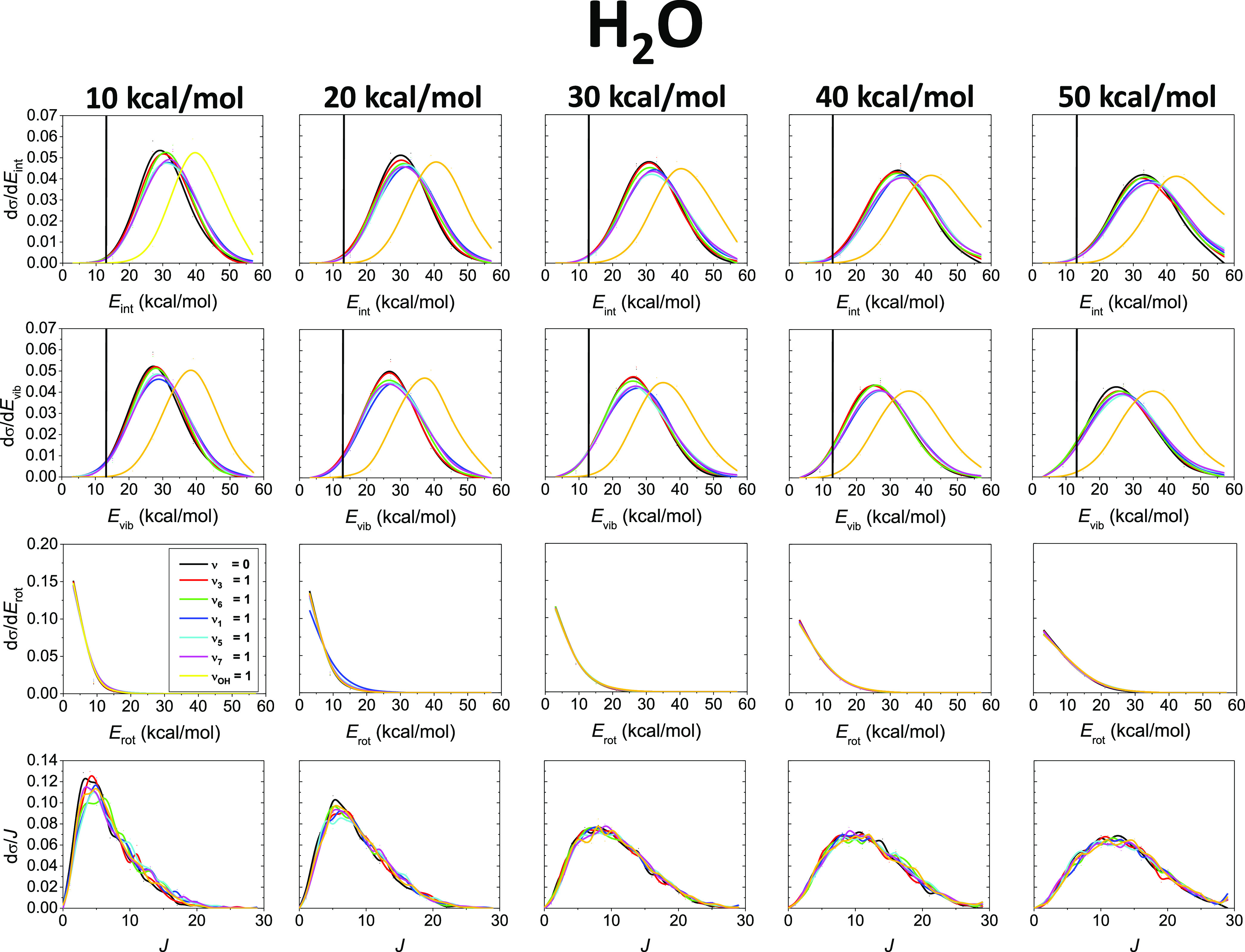
Normalized internal energy (*E*_int_), vibrational energy (*E*_vib_), rotational energy (*E*_rot_), and rotational quantum number (*J*) value distributions for the H_2_O product of the OH(ν_OH_ = 0) + C_2_H_6_(ν_*x*_ = 0, 1), *x* = 3, 6, 1, 5, 7, and OH(ν_OH_ = 1) + C_2_H_6_(ν = 0) reactions at different collision energies. Vertical lines mark the ZPE of the H_2_O molecule. Note that the H_2_O *E*_vib_ distributions for the ground-state reaction (ν = 0) slightly differ from those reported in ref (32) because previously these results were plotted with an incorrect bin size, which is corrected in the present work.

**Figure 5:**
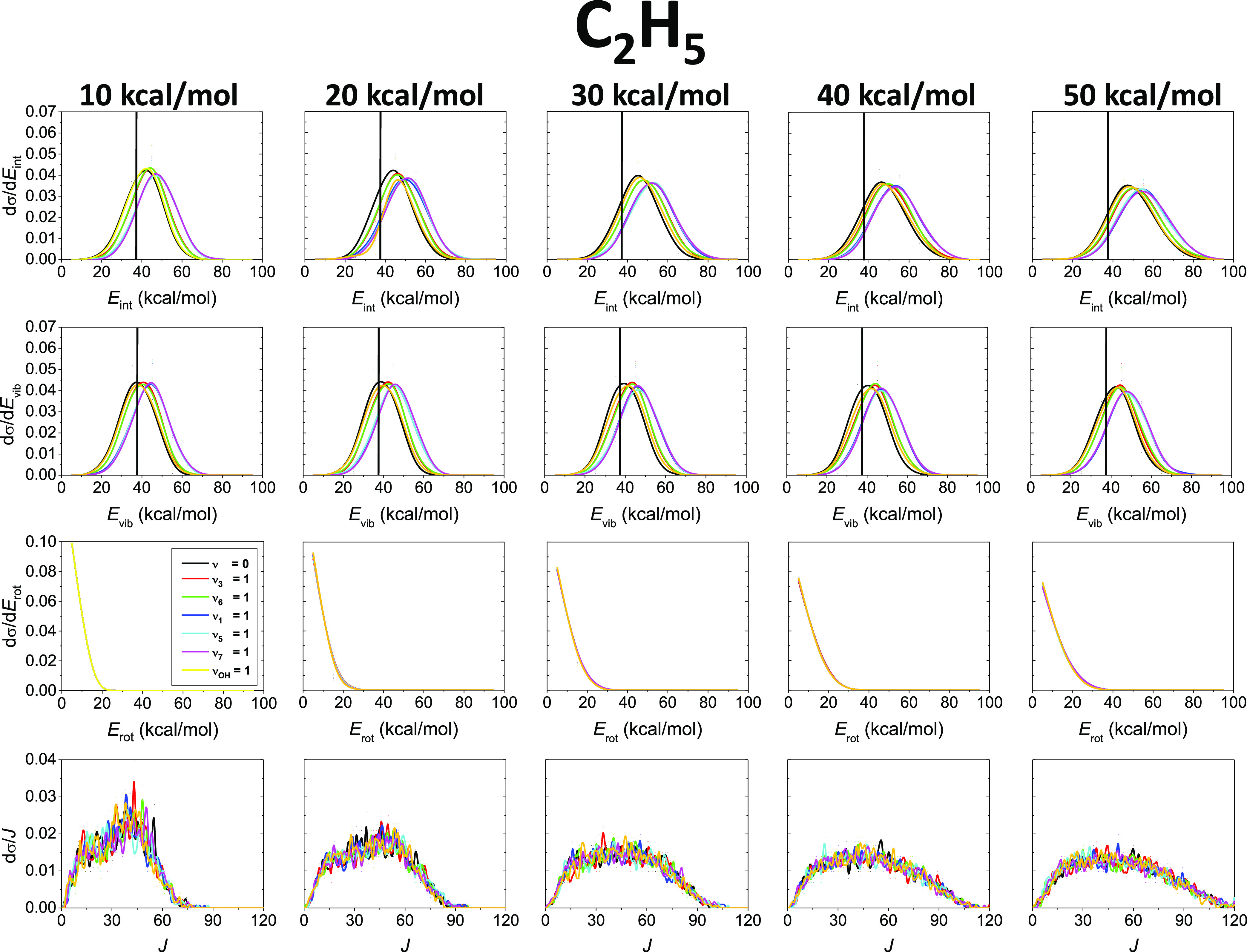
Normalized internal energy (*E*_int_), vibrational energy (*E*_vib_), rotational energy (*E*_rot_), and rotational quantum number (*J*) value distributions for the C_2_H_5_ product of the OH(ν_OH_ = 0) + C_2_H_6_(ν_*x*_ = 0, 1), *x* = 3, 6, 1, 5, 7, and OH(ν_OH_ = 1) + C_2_H_6_(ν = 0) reactions at different collision energies. Vertical lines mark the ZPE of the C_2_H_5_ molecule.

Figures 4 and 5 represent the internal, vibrational, and rotational energy distributions as well as the rotational quantum number distributions at different collision energies for the H_2_O and C_2_H_5_ products, respectively. In the case of the H_2_O molecule, the internal and vibrational energy distributions clearly show mode-specific features because when we excite the OH normal mode these distributions shift toward higher energies. As shown in Figure 4, the energy shifts are around 10 kcal/mol at every *E*_coll_, which is close to excitation energy of the OH reactant, suggesting that the OH stretching is a spectator in the title reaction or, at least, the initial OH vibrational energy remains in the H_2_O product. The energy transfer from the excited CH stretching modes is less predictable based on chemical intuition. Figure 5 shows that the internal and vibrational energy distributions blue-shift upon reactant CH stretching excitations, and most of the initial vibrational energy of C_2_H_6_ flows into the C_2_H_5_ product internal motions, in accord with our conclusions in the discussion of the translational energy distributions. The H-abstraction reaction produces vibrationally excited H_2_O molecules, especially when we excite the initial OH stretching. Only a slight ZPE violation is observable in the case of the H_2_O internal and vibrational energy distributions, which is in contrast with the distributions of the C_2_H_5_ molecule, where the ZPE violation is more significant. For both products, the internal and vibrational energy distributions are widened just a bit and their peaks are not shifted as the collision energy increases. This finding suggests inefficient collision energy transfer to the internal degrees of freedom of the products, in accordance with the significant *E*_coll_ dependence of the translational energy distributions (Figure 3). For both H_2_O and C_2_H_5_, the rotational energy as well as the rotational quantum number value distributions become sensibly broader and shift toward higher energies as *E*_coll_ increases. Significant mode-specific features cannot be found in the case of the rotational distributions. The *J* values go up to 20–30 for H_2_O and 90–120 for C_2_H_5_, and the difference can be explained by the much greater moments of inertia of C_2_H_5_ compared to those of H_2_O.

**Figure 6:**
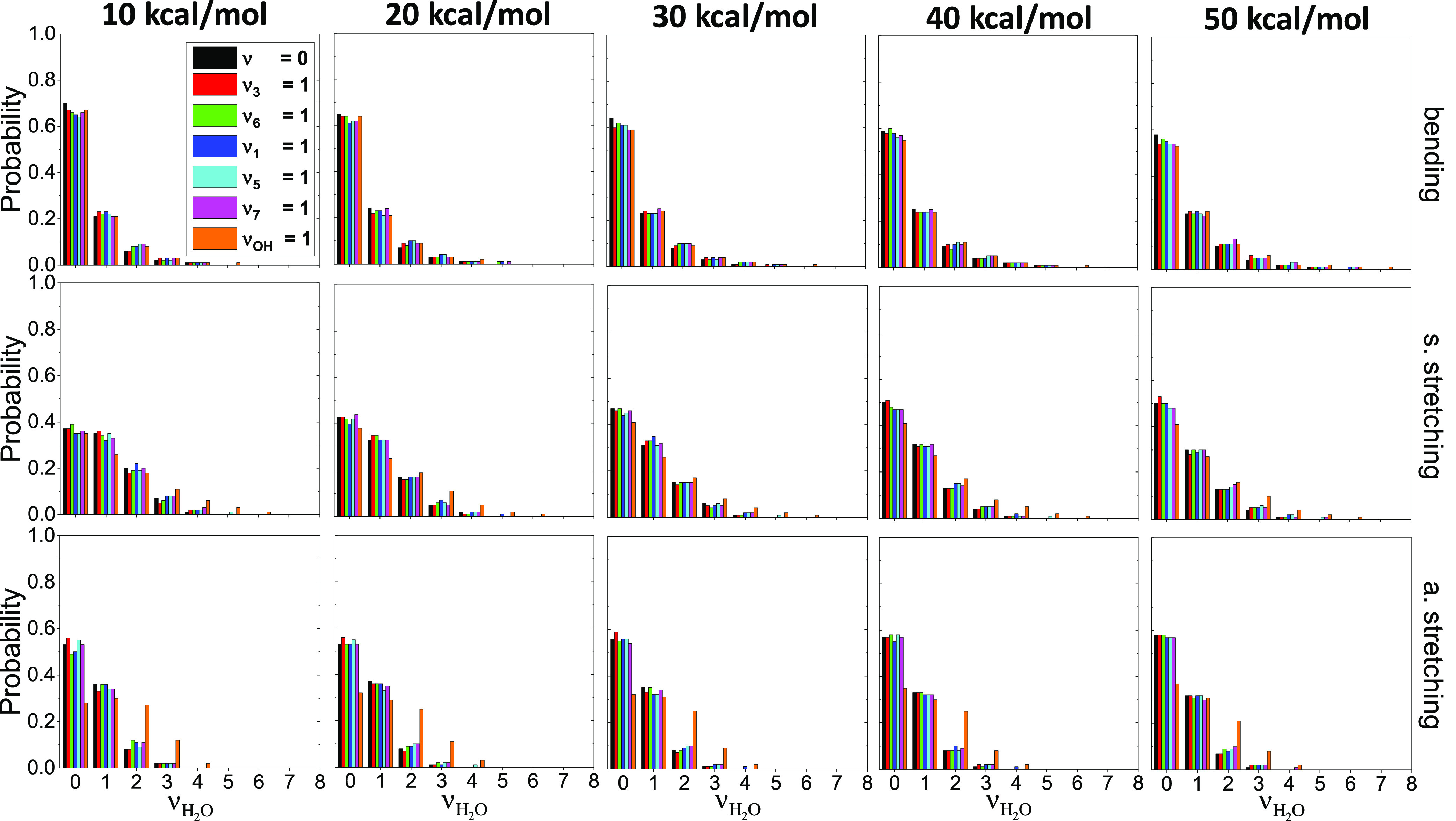
Mode-specific vibrational state distributions for the H_2_O product of the OH(ν_OH_ = 0) + C_2_H_6_(ν_*x*_ = 0, 1), *x* = 3, 6, 1, 5, 7, and OH(ν_OH_ = 1) + C_2_H_6_(ν = 0) reactions at different collision energies. The distributions are normalized for each mode and integrated over the other two modes.

The mode-specific populations of the vibrational states of the H_2_O molecule are shown in Figure 6. In the case of bending and asymmetric stretching, the ground state is the dominant one, and in the case of symmetric stretching at the lowest collision energy, the ground and first excited state is almost equally populated (around 38%) and as the collision energy increases, the ground state becomes more and more promoted. When we initially excite the OH bond, mode-specific feature can be seen in the asymmetric stretching mode distributions, especially in the case of the ν = 2 and ν = 3 vibrational states, i.e., the population of ν = 0 drops and ν = 2 and 3 increase. In all types of motions of the H_2_O molecule, the vibrational states are typically excited with 0, 1, 2, 3, or 4 quanta. These mode-specific product distributions allow direct comparison with experiment.^29^ For the OH(ν_OH_ = 0) + C_2_H_6_(ν = 0) reaction, Butkovskaya and Setser measured the populations of the bending and stretching modes of the H_2_O product at 298 K by analyzing infrared chemiluminescence spectra.^29^ For the bending mode, the measured populations are 67, 25, 6, 2, and 1% for ν = 0, 1, 2, 3, and 4, respectively, in excellent agreement with the present computed values of about 58–70, 21–25, 6–10, 2–4, and 1–2%, in order, without any significant collision energy dependence (see Figure 6). For the stretching modes, only the cumulative distributions were determined experimentally, i.e., 21, 65, and 14% for ν_1,3_ = 0, 1, and 2, respectively, where ν_1,3_ is the sum of the symmetric and asymmetric stretching quanta. Our simulations provide 2–11, 30–42, 29–43, and 18–25% populations, depending on *E*_coll_, for ν_1,3_ = 0, 1, 2, and ≥3, respectively, overestimating the extent of the stretching excitations compared to the measured values. The corresponding hard ZPE-constrained results are 2–8, 39–51, 30–45, and 11–15%, respectively, in slightly better agreement with experiment.

## 4 Summary and Conclusions

We have performed vibrational mode-specific QCT computations for the OH + C_2_H_6_ H-abstraction reaction using a recently developed^32^ analytical full-dimensional ab initio PES. CH stretching excitations clearly enhance the reactivity, CC stretching excitation slightly inhibits the reaction, and OH excitation does not have a significant effect on the cross sections. The comparison of the effects of collision energy and vibrational excitations shows that the OH and CC stretching modes are less effective than translational energy to activate the reaction as expected based on chemical intuition. However, CH stretching excitations have similar or even slightly larger effects on the reactivity than the translational energy, contradicting the Polanyi rules in the case of the early-barrier title reaction. The above findings are based on the non- and soft-constrained trajectories; the hard ZPE constraint slightly changes some of these conclusions. On one hand, here we note that ZPE constraints, especially the hard one, may have different effects on the ground- and excited-state reactions, thus the non-constrained analysis may provide the most realistic vibrational enhancement factors. On the other hand, ZPE constraints may improve the product state populations. Scattering angle distributions are isotropic with slight backward preference at the lowest *E*_coll_ and become more and more forward/stripping preferred as *E*_coll_ increases. The reactivity is the highest if OH attacks from the side-on direction whereas C_2_H_6_ behaves like a spherical object. Product translational energy distributions blue-shift and peak closer and closer to the maximum available energy with increasing collision energy, indicating that most of the initial translational energy transfers to product recoil, and the reaction becomes more and more direct as *E*_coll_ increases. The angular and translational energy distributions do not show any significant mode specificity. The initial OH excitation energy mainly remains in the H_2_O product vibration, whereas the vibrational energy of the ethane modes transfers into the C_2_H_5_ product, even in the case of the reactive CH stretching modes. However, the rotational distributions do not depend on the vibrational states of the reactants. The mode-specific H_2_O vibrational distributions show that the initial OH stretching vibration excites the asymmetric stretching mode of the H_2_O product. The populations of the bending mode decrease with increasing vibrational quantum number, in almost quantitative agreement with available experimental data.^29^ For the stretching modes experimentally, only the cumulative populations were probed, which are overestimated by the present simulations. Nevertheless, both theory and experiment show that H_2_O stretching modes are usually excited by at least one quantum as expected for an early-barrier H abstraction by the OH radical. We hope that the present detailed mode-specific study of the OH + C_2_H_6_ reaction motivates further experiments and simulations on this or similar systems.

## References

[ref1] Polanyi J. C. Some Concepts in Reaction Dynamics. Science 1987, 236, 680–690. 10.1126/science.236.4802.680. 17748308

[ref2] Yan S.; Wu Y. T.; Zhang B.; Yue X.-F.; Liu K. Do Vibrational Excitations of CHD_3_ Preferentially Promote Reactivity Toward the Chlorine Atom?. Science 2007, 316, 1723–1726. 10.1126/science.1142313. 17588925

[ref3] Czakó G.; Bowman J. M. Dynamics of the Reaction of Methane with Chlorine Atom on an Accurate Potential Energy Surface. Science 2011, 334, 343–346. 10.1126/science.1208514. 22021853

[ref4] Zhang Z.; Zhou Y.; Zhang D. H.; Czakó G.; Bowman J. M. Theoretical Study of the Validity of the Polanyi Rules for the Late-Barrier Cl + CHD_3_ Reaction. J. Phys. Chem. Lett. 2012, 3, 3416–3419. 10.1021/jz301649w. 26290965

[ref5] Schatz G. C.; Colton M. C.; Grant J. L. A Quasiclassical Trajectory Study of the State-to-State Dynamics of H + H_2_O → OH + H_2_. J. Phys. Chem. 1984, 88, 2971–2977. 10.1021/j150658a011.

[ref6] Sinha A.; Hsiao M. C.; Crim F. F. Bond-Selected Bimolecular Chemistry: H + HOD(4*v*_OH_) → OD + H_2_. J. Chem. Phys. 1990, 92, 6333–6335. 10.1063/1.458312.

[ref7] Bronikowski M. J.; Simpson W. R.; Girard B.; Zare R. N. Bond-Specific Chemistry: OD:OH Product Ratios for the Reactions H + HOD(100) and H + HOD(001). J. Chem. Phys. 1991, 95, 8647–8648. 10.1063/1.461243.

[ref8] Zhang D. H.; Light J. C. Mode Specificity in the H + HOD Reaction. Full-Dimensional Quantum Study. J. Chem. Soc., Faraday Trans. 1997, 93, 691–697. 10.1039/a605888d.

[ref9] Jiang B.; Guo H. Control of Mode/Bond Selectivity and Product Energy Disposal by the Transition State: X + H_2_O (X = H, F, O(^3^P), and Cl) Reactions. J. Am. Chem. Soc. 2013, 135, 15251–15256. 10.1021/ja408422y. 24044369

[ref10] Song H.; Guo H. Vibrational and Rotational Mode Specificity in the Cl + H_2_O → HCl + OH Reaction: A Quantum Dynamical Study. J. Phys. Chem. A 2015, 119, 6188–6194. 10.1021/acs.jpca.5b03740. 25988486

[ref11] Yoon S.; Henton S.; Zivkovic A. N.; Crim F. F. The Relative Reactivity of the Stretch-Bend Combination Vibrations of CH_4_ in the Cl(^2^P_3/2_) + CH_4_ Reaction. J. Chem. Phys. 2002, 116, 10744–10752. 10.1063/1.1476318.

[ref12] Zhang W.; Kawamata H.; Liu K. CH Stretching Excitation in the Early Barrier F + CHD_3_ Reaction Inhibits CH Bond Cleavage. Science 2009, 325, 303–306. 10.1126/science.1175018. 19608914

[ref13] Meng F.; Yan W.; Wang D. Quantum Dynamics Study of the Cl + CH_4_ → HCl + CH_3_ Reaction: Reactive Resonance, Vibrational Excitation Reactivity, and Rate Constants. Phys. Chem. Chem. Phys. 2012, 14, 13656–13662. 10.1039/c2cp41917c. 22964797

[ref14] Welsch R.; Manthe U. Communication: Ro-Vibrational Control of Chemical Reactivity in H + CH_4_ → H_2_ + CH_3_: Full-Dimensional Quantum Dynamics Calculations and a Sudden Model. J. Chem. Phys. 2014, 141, 051102 10.1063/1.4891917. 25106559

[ref15] Qi J.; Song H.; Yang M.; Palma J.; Manthe U.; Guo H. Communication: Mode Specific Quantum Dynamics of the F + CHD_3_ → HF + CD_3_ Reaction. J. Chem. Phys. 2016, 144, 171101 10.1063/1.4948547. 27155615

[ref16] Fu B.; Shan X.; Zhang D. H.; Clary D. C. Recent Advances in Quantum Scattering Calculations on Polyatomic Bimolecular Reactions. Chem. Soc. Rev. 2017, 46, 7625–7649. 10.1039/C7CS00526A. 29143835

[ref17] Jiang B.; Guo H. Relative Efficacy of Vibrational vs. Translational Excitation in Promoting Atom-Diatom Reactivity: Rigorous Examination of Polanyi’s Rules and Proposition of Sudden Vector Projection (SVP) Model. J. Chem. Phys. 2013, 138, 234104 10.1063/1.4810007. 23802948

[ref18] Guo H.; Jiang B. The Sudden Vector Projection Model for Reactivity: Mode Specificity and Bond Selectivity Made Simple. Acc. Chem. Res. 2014, 47, 3679–3685. 10.1021/ar500350f. 25393632

[ref19] Corchado J. C.; Chamorro M. G.; Rangel C.; Espinosa-Garcia J. State-to-State Dynamics of the Cl(^2^P) + C_2_H_6_(ν_5_, ν_1_ = 0, 1) → HCl(*v*′, *j*′) + C_2_H_5_ Hydrogen Abstraction Reactions. Theor. Chem. Acc. 2019, 138, 26 10.1007/s00214-019-2416-3.

[ref20] Papp D.; Li J.; Guo H.; Czakó G. Vibrational Mode-Specificity in the Dynamics of the Cl + C_2_H_6_ → HCl + C_2_H_5_ Reaction. J. Chem. Phys. 2021, 155, 114303 10.1063/5.0062677. 34551541

[ref21] Papp D.; Czakó G. Vibrational Mode-Specific Dynamics of the F(^2^P_3/2_) + C_2_H_6_ → HF + C_2_H_5_ Reaction. J. Chem. Phys. 2021, 155, 154302 10.1063/5.0069658. 34686045

[ref22] Lu D.; Li J. Mode Specificity of a Multi-Channel Reaction Prototype: F + CH_3_OH → HF + CH_3_O/CH_2_OH. Theor. Chem. Acc. 2020, 139, 157 10.1007/s00214-020-02671-3.

[ref23] Lu D.; Li J. Mode Specificity Dynamics of Prototypical Multi-Channel H + CH_3_OH Reaction on Globally Accurate Potential Energy Surface. Chin. J. Chem. Phys. 2022, 35, 481–487. 10.1063/1674-0068/cjcp2201018.

[ref24] Yin C.; Czakó G. Theoretical Vibrational Mode-Specific Dynamics Studies for the HBr + C_2_H_5_ Reaction. Phys. Chem. Chem. Phys. 2023, 25, 3083–3091. 10.1039/D2CP05334A. 36620947

[ref25] Yin C.; Czakó G. Vibrational Mode-Specific Quasi-Classical Trajectory Studies for the Two-Channel HI + C_2_H_5_ Reaction. Phys. Chem. Chem. Phys. 2023, 25, 9944–9951. 10.1039/D2CP05993B. 36951419

[ref26] Baulch D. L.; Craven R. J. B.; Din M.; Drysdale D. D.; Grant S.; Richardson D. J.; Walker A.; Watling G. Rates of Hydroxy Radical Reactions with Methane, Ethane and Propane over the Temperature Range 403–696 K. J. Chem. Soc., Faraday Trans. 1 1983, 79, 689–698. 10.1039/f19837900689.

[ref27] Tully F. P.; Droege A. T.; Koszykowski M. L.; Melius C. F. Hydrogen-Atom Abstraction from Alkanes by OH. 2. Ethane. J. Phys. Chem. 1986, 90, 691–698. 10.1021/j100276a042.

[ref28] Senosiain J. P.; Musgrave C. B.; Golden D. M. Use of Quantum Methods with Transition State Theory: Application to H-Atom Metathesis Reactions. J. Phys. Chem. A 2001, 105, 1669–1675. 10.1021/jp002424l.

[ref29] Butkovskaya N. I.; Setser D. W. Infrared Chemiluminescence from Water-Forming Reactions: Characterization of Dynamics and Mechanisms. Int. Rev. Phys. Chem. 2003, 22, 1–72. 10.1080/0144235021000033381.

[ref30] Rangel C.; Garcia-Chamorro M.; Corchado J. C.; Espinosa-Garcia J. Kinetics and Dynamics Study of the OH + C_2_H_6_ → H_2_O + C_2_H_5_ Reaction Based on an Analytical Global Potential Energy Surface. Phys. Chem. Chem. Phys. 2020, 22, 14796–14810. 10.1039/D0CP02776F. 32578642

[ref31] Gruber B.; Czakó G. Benchmark ab Initio Characterization of the Abstraction and Substitution Pathways of the OH + CH_4_/C_2_H_6_ Reactions. Phys. Chem. Chem. Phys. 2020, 22, 14560–14569. 10.1039/D0CP02560G. 32596706

[ref32] Gruber B.; Tajti V.; Czakó G. Full-Dimensional Automated Potential Energy Surface Development and Dynamics for the OH + C_2_H_6_ Reaction. J. Chem. Phys. 2022, 157, 074307 10.1063/5.0104889. 35987568

[ref33] Győri T.; Czakó G. Automating the Development of High-Dimensional Reactive Potential Energy Surfaces with the Robosurfer Program System. J. Chem. Theory Comput. 2020, 16, 51–66. 10.1021/acs.jctc.9b01006. 31851508

[ref34] Hase W. L. Encyclopedia of Computational Chemistry; Wiley: New York, 1998; pp 399–407.

[ref35] Czakó G. Gaussian Binning of the Vibrational Distributions for the Cl + CH_4_(*v*_4/2_ = 0, 1) → H + CH_3_Cl(*n*_1_*n*_2_*n*_3_*n*_4_*n*_5_*n*_6_) Reactions. J. Phys. Chem. A 2012, 116, 7467–7473. 10.1021/jp3044797. 22721354

[ref36] Li J.; Jiang B.; Guo H. Reactant Vibrational Excitations Are More Effective than Translational Energy in Promoting an Early-Barrier Reaction F + H_2_O → HF + OH. J. Am. Chem. Soc. 2013, 135, 982–985. 10.1021/ja311159j. 23301908

[ref37] Song H.; Guo H. Mode Specificity in the HCl + OH → Cl + H_2_O Reaction: Polanyi’s Rules vs Sudden Vector Projection Model. J. Phys. Chem. A 2015, 119, 826–831. 10.1021/jp512021m. 25580616

[ref38] Xiang H.; Lu Y.; Song H.; Yang M. Mode-Specific Quantum Dynamics Study of OH + H_2_S → H_2_O + SH Reaction. Chin. J. Chem. Phys. 2022, 35, 200–206. 10.1063/1674-0068/cjcp2112278.

